# Exploring the knowledge, attitudes, and performance of dentists in providing care to elderly patients

**DOI:** 10.1186/s12903-023-03832-z

**Published:** 2024-01-09

**Authors:** Fateme Najmi Nouri, Mehrnaz Karimi Afshar, Marzieh Karimi Afshar, Hamze Hooshmand, Rahil Ghorbani Nia

**Affiliations:** 1 Department of Community Dentistry, Faculty of Dentistry, Kerman University of Medical Sciences, Kerman, Iran; 2 Department of Prosthodontics, Faculty of Dentistry, Shiraz University of Medical Sciences, Shiraz, Iran; 3 Department of Orthodontics, Faculty of Dentistry, Kerman University of Medical Sciences, Kerman, Iran; 4 Private Practice, Kerman, Iran; 5 Health Care Management, Noncommunicable Diseases Research Center, Noncommunicable Diseases Research Center, Bam University of Medical Sciences & Health Services, Shahid Rajaie Blvd, Bam, Iran

**Keywords:** Oral health, Geriatric dentistry, Knowledge, Attitude, Performance

## Abstract

**Background:**

Supportive care and dental treatment for older adults are crucial in addressing the global emergency of population aging, requiring specialized healthcare services and knowledge-based practices.

**Methods:**

This cross-sectional content analysis study was conducted on 150 general dentists in Kerman in 2021. The participants were selected using cluster sampling. The data were collected using a questionnaire with four sections assessing the participants’ demographic characteristics, knowledge, attitudes, and performance. The data were analyzed with SPSS-26 software using the t-test, ANOVA, and linear regression analysis.

**Results:**

The dentists’ mean age was 36.10 ± 7.60 years. The mean knowledge score of the participants was 5.29 ± 1.49 (out of 9). The mean attitude score was 59.42 ± 11.6 (out of 85), and the mean performance score was 24.13 ± 4.96 (out of a maximum of 35). The data showed a positive relationship between the dentists’ knowledge, attitudes, and performance. However, the participants’ gender had no significant correlation with their knowledge, attitudes, or performance. It was also shown that 50% of dentists had adequate experience treating elderly patients with complex medical problems.

**Conclusions:**

The participants had an adequate level of knowledge and performance and a positive attitude toward dental care for older adults. Health officials and administrators need to organize and hold effective training and refresher courses on geriatric dentistry to promote dentists’ knowledge and performance.

**Trial registration:**

Ethics code IR.KMU.REC.1401.007.

## Background

Population aging is one of the biggest global accomplishments and the outcome of technological growth, improved healthcare, diagnosis, and treatment, and increased life expectancy [1]. Improving public health and safety is one of the important missions of the health system, and this system can adopt a new approach to replacing unwanted care and services with goals and plans that value old age. Besides, the health system should follow integrated strategies to improve older adults’ health and well-being [2]. Given the current increasing trend of the elderly population, the population of this group in developing countries will make up 80% of the world’s elderly population by 2050 [3]. Currently, 2.8% of Iran’s population is made up of older adults over 60 years old, and this figure is predicted to reach 26% in less than 4 decades [4].

Aging is associated with the occurrence of some chronic diseases, most of which have oral manifestations that may lead to chewing and swallowing problems [5]. Furthermore, aging is associated with many physiological changes in the oral cavity, including the loss of tooth translucency and surface details and dental wear and erosion [6]. A study showed that oral and dental health is somewhat neglected in older adults because these people need care for their daily activities such as eating, taking medication, dressing, bathing, general healthcare, and physical therapy. As a result, less time is spent on activities that are usually less important to older adults, including oral care [7]. Moreover, financial restrictions, a lack of family support or transportation problems, and the unavailability of dental services can have harmful effects on the comfort, beauty, speech, chewing, and consequently the quality of life of older adults [8].

The World Health Organization has emphasized older adults’ need for adequate access to quality dental services [9]. However, dentists, who are responsible for maintaining the public’s oral health, have problems providing services to older adults. One of the most important issues is the lack of knowledge and practice in similar situations. Moreover, it has been shown that some factors, such as gender, age, and experience of the dentist, as well as the number of patients over 75 years of age visited by each dentist, can play a major role in reducing these problems [10]. The difficulties of performing dental treatment for older adults and treating various systemic diseases in these patients require adequate knowledge about correct behavior and an effective treatment plan for these patients. Accordingly, the present study aims to explore the knowledge, attitudes, and performance of dentists in providing care to elderly patients in Kerman in 2021.

## Methods

### Study design

This descriptive-analytical cross-sectional study was conducted on the dentists working in healthcare centers in five central, eastern, western, northern, and southern urban districts in Kerman in 2021. The dentists who had work experience of less than one year, dental students, and those who were not willing to participate in the study were excluded from the study.

### Sample size and sampling method

Given the total number of general dentists working in the offices of health centers in Kerman, 150 dentists were selected using multistage random sampling. To increase the dispersion and accuracy of the data, and to address the cultural and social characteristics of the population of different regions of the city, the city was divided into five central, eastern, western, northern, and southern districts. Then, the dental clinics in each district were selected for sampling. Afterward, the participants were selected randomly based on the number of people under coverage in each clinic. Taking the 95% confidence interval, 0.5 error level, and 0.04 sampling accuracy, the sample size was estimated using the following formula [11]:

$${\text{n}} = \frac{{{{\left( {{Z_{1 - \frac{\alpha }{2}}}} \right)}^2}\rho \left( {1 - \rho } \right)}}{{{{\text{d}}^2}}}$$

$${Z_{1 - \frac{\alpha }{2}}} = 1.96\quad \quad \quad \quad \quad \quad P < 0.05$$

### Data collection tool

The instrument used for data collection was a questionnaire consisting of two sections: The items in the first section assessed the participants’ demographic information, including gender, age, marital status, working hours, visits to elderly patients, the number of visits to elderly patients in the last week, and whether they had completed the geriatric dentistry course at the college. The second section measured the participants’ attitudes (17 items), performance (7 items), and knowledge (9 items). The items were scored on a 5-point Likert scale (strongly disagree = 1 to strongly agree = 5). Some items were also scored reversely. The minimum and maximum scores for the participants’ attitudes were 17 and 85, respectively. The participants’ performance was measured using items scored on a Likert scale ranging from strongly disagree to strongly agree, in a score range of 7 to 35. The participants’ knowledge was measured using items answered true, false, or I don’t know. A correct answer was scored 1, and the false answers and I don’t know were scored 0. The minimum and maximum scores for the participants’ knowledge were 0 and 9, respectively. The validity and reliability of the items were confirmed in previous studies [12–14].

### Statistical analysis

The collected data were analyzed using the t-test, ANOVA, Pearson correlation and regression analysis with SPSS software (version 26). A significance level of 0.05 was considered for data analysis.

## Results

Most of the participants were male (60%) and married (66%). Moreover, 66% of them had passed geriatric dentistry courses, and 65.3% of them had an older adult in the family. A majority of the participants (92.6%) stated that they had visited a nursing home, and some of them did not have daily visits from elderly patients (59.3%). In addition, 92.7% of the participants had visited elderly patients in the last week. The participants’ average age was 36.10 ± 7.60 years. Table 1 displays the participants’ demographic data: Table 1.

**Table 1:** The frequency distribution of the participants’ demographic characteristics

Variable	Category	Number	Percentage	
Gender	Male	90	60	
	Female	60	40	
Marital status	Single	51	34	
	Married	99	66	
Geriatric dentistry courses completed?	Yes	99	66	
	No	51	34	
Having older adults in the family	Yes	98	65.3	
	No	52	34.7	
Visiting nursing homes	Yes	139	92.6	
	No	11	7.4	
Daily visits from elderly patients	Yes	61	40.7	
	No	89	59.3	
Visited elderly patients in the last week	Yes	139	92.7	
	No	11	7.3	
Number of healthcare centers in urban districts	Central	50	33.3	
	Northern	34	22.6	
	Southern	23	15.3	
	Eastern	20	13.3	
	Western	23	15.3	
Job experience (year)	< 5	34	22.6	
	5–10	44	29.4	
	> 10	72	48	
**Variable**	**Mean**	**SD**	**Max**	**Min**
Age	36.10	7.60	55	26
Job experience	9.24	7.04	35	1
Graduation time	9.02	6.85	27	1
Weekly working hours	35.34	10.67	72	6
Older adults visited in the last week	7.29	7.52	40	0

### Geriatric oral health-related knowledge

The participants’ answers to knowledge questions are shown in Table 2. As can be seen, 84% of the participants gave a correct answer to the question “Root decay increases with age”. Besides, the percentage of correct answers to the statement “The prevalence of periodontal diseases increases with aging” was 74%. In addition, 66% of the participants correctly indicated that “most cases of dry mouth in older adults are directly related to the physiological changes of aging”. The least frequent correct answers (38%) were given to the item “Dislocation of the TMJ joint is one of the common complications of old age,” followed by the item “The use of local anesthesia with epinephrine is not restricted for older adults who use digoxin,” with 38.7% of the correct answers (Table 2).

**Table 2:** The frequency of the responses given by the dentists to the knowledge questions

Row	Item	Incorrect		Correct		I don’t know	
		Frequency	%	Frequency	%	Frequency	%
1	The use of NSAIDs increases the risk of bleeding for Alzheimer’s patients who use anticholinesterase drugs (a common drug group in the treatment of Alzheimer’s).	31	20.7	83	55.3	36	24.0
2	Dislocation of the TMJ joint is one of the common complications of old age.	71	46.7	57	38.0	22	15.3
3	The use of local anesthesia with epinephrine is not restricted to older adults who use digoxin.	64	42.7	58	38.7	28	18.7
4	The probability of aspiration during dental treatment is higher in patients with Parkinson’s disease.	52	34.7	75	50.0	23	15.3
5	Most cases of dry mouth in older adults are directly related to the physiological changes of old age.	36	24.0	100	66.7	14	9.3
6	People with end stages of dementia are nervous and aggressive in the dental office.	59	39.3	65	43.3	26	17.3
7	The prevalence of periodontal diseases increases with old age.	31	20.7	111	74.0	81	54
8	The tasting ability does not change much with age.	81	54.0	59	39.3	5	3.3
9	Root decay increases with age.	19	12.7	126	84.0	5	3.4

### Attitudes toward old age and the oral health of older adults

The data showed that 34.7% of the participants strongly agreed with the statement “It is the duty of the community to care for and support older adults” and 27.3% strongly agreed with the statement “In the future, general dentists should have the ability to treat older adults to provide the best possible care for the elderly population”. The participants’ answers to the attitude questions are shown in Table 3.

**Table 3:** The frequency of the responses given by the dentists to the attitude questions

Row	Item	Strongly disagree	Disagree	Undecided	Agree	Strongly agree
		%	%	%	%	%
1	It’s pleasant to spend time with most older adults.	1.3	22.7	18.7	46.7	10.7
2	If I have a choice, I prefer to visit younger patients more than older adults.	2.0	15.3	20.7	51.3	10.7
3	The community has to care for and support older adults.	0.7	12.0	5.3	47.3	34.7
4	Providing medical care for older adults consumes too many human and material resources.	10.7	49.3	14.7	14.0	11.4
5	As people get older, they become more confused.	4.0	40.7	13.3	35.3	6.7
6	Elderly patients appreciate the medical care I provide more than younger patients.	1.3	20.7	20.7	39.3	18.0
7	Obtaining a medical history from elderly patients is often a challenge	14.7	49.3	12.0	18.7	5.3
8	I tend to have more attention and sympathy towards my elderly patients (than young patients).	2.0	12.7	22.7	45.3	17.4
9	Older adults generally do not contribute much to the community.	18.7	56.7	12.7	10.0	2.0
10	Treatment of elderly patients with chronic diseases is hopeless.	10.7	38.0	30.0	20.7	0.7
11	Older adults cannot pay enough for their health expenses.	10.0	43.3	10.0	26.0	11.0
12	Older adults are generally kike speed bumps in modern society.	18.0	46.0	22.7	8.7	4.6
13	It is interesting to listen to older people’s accounts of their past experiences.	3.3	16.7	5.3	50.0	24.7
14	Older adults do not add much to the community.	20.0	52.0	14.0	8.7	5.3
15	A part of the health expenses of older adults should be spent by the government on pediatric patients and AIDS research.	42.7	34.7	6.7	8.0	8.0
16	In the future, general dentists must have the ability to treat older adults to provide the best possible care for the elderly population.	1.3	18.7	18.7	34.0	27.3
17	Prevention of oral diseases in older adults is not economically profitable for the dentist.	8.3	47.3	25.3	11.3	7.4

### Performance related to older adults’ oral and dental health

Table 4 shows the participants’ responses to the performance questions. As can be seen, 54.7% of the participants agreed or strongly agreed that they communicate well with older adults. In addition, 66.6% of the participants stated that they could express empathy with and understand elderly patients. The data also indicated that 18.7% of the participants strongly agreed that they had received sufficient education in geriatric dentistry in college (Table 4).

**Table 4:** The frequency of the responses given by the dentists to the performance questions

Row	Item	Strongly disagree	Disagree	Undecided	Agree	Strongly agree
		%	%	%	%	%
1	I can prepare an effective treatment plan based on the needs and preferences of elderly patients.	0.7	37.3	13.3	32.0	16.7
2	I can communicate well with elderly patients.	-	6.0	39.3	38.0	16.7
3	I can provide preventive care and treatment for elderly patients.	0.7	25.3	22.7	36.7	14.7
4	I can manage medical emergencies of elderly patients.	5.3	10.7	29.3	40.0	14.7
5	I can empathize with and understand elderly patients.	-	16.0	17.3	45.3	21.3
6	I have enough experience to manage the complexities of treating elderly patients	4.0	20.0	16.0	36.7	13.3
7	I have received adequate education in geriatric dentistry at university.	9.3	21.3	20.0	30.7	18.7

The mean scores for the participants’ attitudes, performance, and knowledge were 59.42 ± 6.11, 24.13 ± 4.96, and 5.29 ± 1.49, respectively. In addition, the total score of the questionnaire was reported as 89.77 ± 8.96.

Pearson’s correction test showed a positive and significant relationship between the participants’ performance and the number of elderly visits. The participants who treated and visited more older adults had a better performance. A significant and positive relationship was observed between the participants’ knowledge, working hours, and graduation time. The total score of the questionnaire did not show a statistically significant relationship with any of the mentioned variables. More details are shown in Table 5.

**Table 5:** The correlations between the participants’ knowledge, attitudes, and performance and their demographic variables

Variable	Statistics	Age	Visiting nursing homes	Weekly working hours	Graduation time	Older adults visited in the last week
Attitudes	Pearson’s correlation	0.69	0.082	-0.076	0.003	-0.004
	P-value	0.425	0.347	0.385	0.972	0.965
Performance	Pearson’s correlation	0.113	0.036	-0.029	-0.028	0.245
	P-value	0.182	0.671	0.731	0.737	*0.002
Knowledge	Pearson’s correlation	0.131	0.151	0.245	0.193	0.098
	P-value	0.125	0.079	*0.004	*0.024	0.254
Total score	Pearson’s correlation	0.122	0.088	-0.026	-0.049	0.152
	P-value	0.172	0.326	0.770	0.588	0.089

*Significance indicated

Based on the results of t-test, Table 6 shows the relationship between the participants’ gender, marital status, taking geriatric dentistry courses in college, having older adults in the family, and visiting nursing homes with knowledge, attitudes, and performance (Table 6).

**Table 6:** The relationship between the participants’ demographic variables and their attitude, performance, and knowledge

Variable		Category	Mean	SD	P-value
Attitudes	Gender	Male	60.21	5.90	0.222
		Female	58.90	6.33	
	Marital status	Single	60.48	6.49	0.054
		Married	58.36	5.08	
	Geriatric dentistry courses completed?	Yes	59.57	7.23	0.929
		No	59.46	5.12	
	Having older adults in the family	Yes	60.10	6.48	0.603
		No	59.53	5.60	
	Visiting nursing homes	Yes	60.00	5.97	0.667
		No	59.48	5.88	
Performance	Gender	Male	24.50	5.10	0.270
		Female	23.55	4.77	
	Marital status	Single	24.67	5.19	0.090
		Married	23.16	4.54	
	Geriatric dentistry courses completed?	Yes	26.04	4.72	*0.001
		No	23.15	4.72	
	Having older adults in the family	Yes	25.04	5.36	0.092
		No	23.57	4.68	
	Visiting nursing homes	Yes	23.78	4.48	0.672
		No	24.20	1.50	
Knowledge	Gender	Male	5.45	1.44	0.155
		Female	5.08	1.55	
	Marital status	Single	5.47	1.53	0.078
		Married	5.00	1.39	
	Geriatric dentistry courses completed?	Yes	5.46	1.40	0.407
		No	5.23	1.57	
	Having older adults in the family	Yes	5.41	1.84	0.535
		No	5.23	1.28	
	Visiting nursing homes	Yes	5.24	1.58	0.966
		No	5.24	1.43	

*Significance indicated

Based on findings of t-test results, Table 7 shows the relationship between the participants’ demographic variables and the total score on the questionnaire. As can be seen, the married dentists scored significantly higher than the single dentists (Table 7). In other cases, no statistically significant difference was observed.

**Table 7:** The relationship between the participants’ demographic variables and the total score on the questionnaire

Variable		Category	Mean	SD	P-value
Total score	Gender	Male	90.40	9.30	0.111
		Female	87.70	8.97	
	Marital status	Single	91.10	9.89	*0.003
		Married	86.48	7.20	
	Geriatric dentistry courses completed?	Yes	91.25	9.84	0.077
		No	88.27	8.06	
	Having older adults in the family	Yes	91.31	10.57	0.083
		No	88.34	8.28	
	Visiting nursing homes	Yes	89.21	9.42	0.966
		No	89.13	8.78	

*Significance indicated

An analysis of variance (ANOVA) showed that the dentists who examined and treated more older adults performed significantly better (P = 0.001). Table 8 shows the results of the regression analysis using the backward method. As the data in this table indicated, higher levels of performance and knowledge and fewer working hours had a statistically significant relationship with more positive attitudes. Being married, having less time passed since graduation, passing dentistry courses, having an elderly person in the family, having a greater number of visits, and having more positive attitudes have significant correlations with better performance. Visiting more older adults, having more working hours, and having more positive attitudes have a significant positive relationship with a higher level of knowledge. The total score on the questionnaire had a statistically significant relationship with marital status, and a greater number of older adults visited (Table 8).

**Table 8:** The regression between the participants demographic variables and their attitude, performance, and knowledge

Variable		B	t	P-value
Attitudes	Working hours	-0.134	-2.786	0.006^*^
	Performance	0.249	2.417	0.017^*^
	Knowledge	1.002	7 − 2,7	0.008^*^
Performance	Being married	-1.985	-2.084	0.039^*^
	Graduation time	-0.144	-2.216	0.029^*^
	Passing geriatric dentistry courses	2.420	2.845	0.005^*^
	Having older adults in the family	8 − 1,7	2.054	0.042^*^
	Number of older adults visited	1.274	4.055	0.000^*^
	Attitudes	0.1999	2.772	0.007^*^
Knowledge	Working hours	0.39	3.398	0.001^*^
	Number of older adults visited	0.035	2.136	0.035^*^
	Attitudes	0.630	2.136	0.005^*^
Total score	Being married	-5.437	-2.926	0.004^*^
	Graduation time	-2.227	-1.707	0.091
	Number of older adults visited	0.233	2.091	0.039^*^

**Fig. 1:**
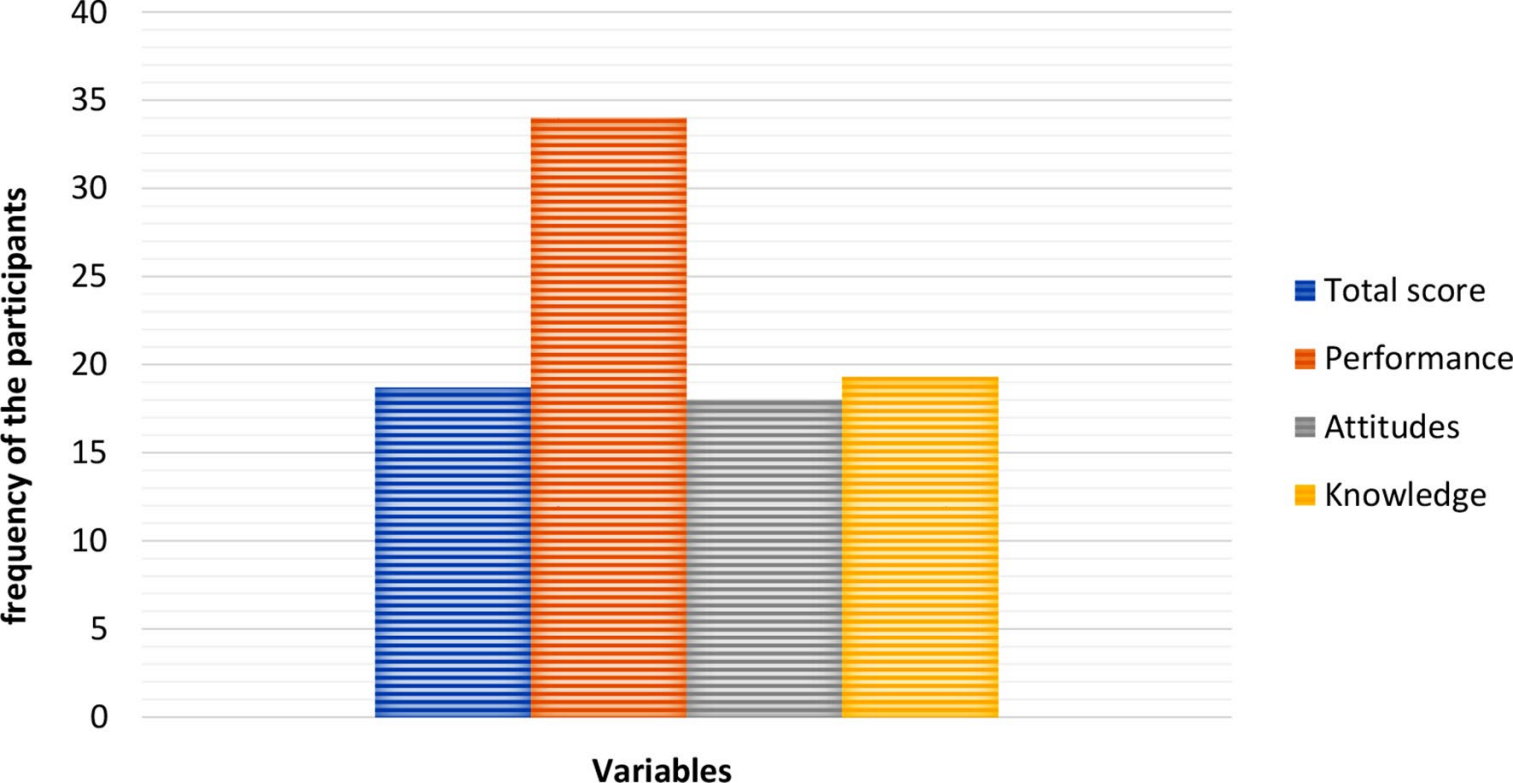
The frequency of the participants in terms of knowledge, attitudes, and performance

Data analysis showed that 18% of the participants had positive attitudes, 34% had good performance, and 19.3% had a high level of knowledge. Besides, the overall score was satisfactory for 18.7% of the participants, as shown in Fig. 1.

## Discussion

The study found that general dentists in Kerman had poor attitudes, knowledge, and performance, affecting their ability to detect and prevent oral health issues in older adults. Regular visits are crucial for addressing these issues [15].

The present study showed that 19.3% of dentists had a high level of knowledge. A study by Alaei et al. [8] conducted on dentists participating in the Dental Congress in Tehran showed that 11.5% of dentists had an average level of knowledge. Similarly, Sargeran et al. [4] reported an average level of knowledge among dentists. Aldhuwayhi (2021) showed that dental students in Saudi Arabia failed to follow a positive approach to providing primary healthcare despite the rapid growth of the elderly population [16]. In their study in Croatia, Madunic et al. showed that dental studies should highlight higher levels of knowledge and skills in the treatment of older adults [17]. Tahani [14] also found that 2.6% of the dentists had a good level of knowledge. Hatami et al. found dental students have limited knowledge of geriatric dentistry. To improve patient care, geriatric dentistry courses should be developed to address the elderly population’s needs, including rehabilitation services for disabled individuals [13].

The present study indicated that 18% of dentists had positive attitudes toward older adults. Alaei et al. [8] reported that 39.7% of dentists had positive attitudes toward older adults. Moreover, Sargeran et al. [4] reported moderate attitudes among dentists. Other studies also reported similar results [15, 18, 19]. Given the increasing trend of the elderly population, dentists must receive sufficient training so that they can easily treat the oral and dental diseases of older adults. Since most of the problems in this field seem to be due to inadequate training and clinical experiences, it is possible to increase dentists’ positive attitudes and improve performance in oral health and care.

The data in the present study revealed that 34% of dentists had good performance. Sargeran et al. [4] reported that dental students had a moderate performance, and Hatami et al. [13] reported that the performance of dental students was low to moderate. Iran’s population structure changes necessitate examining population aging and raising awareness. Effective communication with older adults is crucial for providing oral and dental health services, emphasizing the importance of oral and dental hygiene.

The data in the present study showed a statistically significant relationship between performance and the completion of geriatric dentistry courses. In contrast, Alaei et al. [8] did not report a statistically significant difference between the attitudes and knowledge of dentists who completed or did not complete geriatric dentistry courses. Kossioni et al. [20] evaluated the status of geriatric dental education in some European dental schools and showed that the majority of universities have special modules and courses dedicated to older adults. Tahani et al. found Iran’s geriatric dentistry programs insufficient; teachers must choose effective teaching methods, with traditional and distance education contributing to dental course goals. Virtual education may replace traditional education in the future [14, 21].

In the present study, no statistically significant difference was observed between the dentists’ gender and their knowledge, attitudes, and performance, possibly due to uniform education in the field of geriatric dentistry. Similarly, Tahani [14] reported no significant difference between dentists’ attitudes and gender. However, a study by Bots-VantSpijker et al. [22] in the Netherlands and Belgium showed that female dentists had more positive attitudes toward older adults. These conflicting findings could be attributed to cultural and social factors governing the countries as well as the differences in the sample size.

The present study found no statistically significant relationship between dentists’ age and their knowledge, attitudes, and performance. In a similar vein, Alaei et al. [8] reported that dentists’ age and gender were not associated with knowledge and attitudes. However, this finding was not consistent with the results reported by Sargeran et al. [4], Bots-VantSpijker et al. [23], and De Visschere et al. [24] who reported a significant correlation between dentists’ gender and knowledge. Inconsistent results may be due to participants’ prior geriatrics courses, family involvement, and frequent visits to nursing homes. Refresher courses should incorporate geriatric dentistry materials.

The findings of the present study confirmed a statistically significant relationship between dentists’ knowledge and attitudes. Likewise, Tahani et al. [14] reported a significant correlation between dentists’ knowledge and attitudes. Rapid demographic changes have necessitated the inclusion of geriatric dentistry in the curriculum of many dental schools. There is a need for a greater focus on special clinical skills and changes in the attitudes of dental students toward caring for elderly patients. Geriatric dental education can be defined as “the part of the pre-doctoral curriculum that deals with the specific knowledge, attitudes, and technical skills required in providing oral healthcare for older adults” [25]. Dental schools should train students in dental management so that they are competent and confident in managing the treatment needs of elderly patients.

The study found a significant relationship between dentists’ attitudes and performance. To improve attitudes and treat older adults more effectively, policies and plans should be developed based on their characteristics and needs [26]. Raising awareness, offering training courses, and incorporating ICT training in online education and remote dentistry should be prioritized by health policymakers. Aging is a global accomplishment, and meeting the needs of older adults is crucial for active aging. Accurate needs assessments are necessary for effective planning, and psychological and physical needs should be considered in policies and programs related to elderly health. Dentists have positive attitudes towards older adults, but with the increasing elderly population, it is necessary to hold more refresher programs or workshops on geriatric dentistry [27].

Since the data in this study were collected using self-report questionnaires completed by dentists, some answers could be influenced by social desirability bias, which was out of the researcher’s control. Since the dentists working in Kerman participated in this research project, the results cannot be generalized to all dentists in Iran.

The department’s practical programs should require the training groups to include specialized instruction in elderly dentistry, and students should be required to treat and oversee this patient population at some time throughout their studies. Government, organization, and other governmental faculties, should work together in this manner. They should also consider to enhance dental care in this community and implement the required policies.

## Conclusion

Following the findings of the present study, dentists in Kerman did not have good knowledge, performance, and attitudes toward geriatric dentistry. Thus, since geriatric dentistry courses have a small share in the basic science courses and higher academic levels for dental students, effective training and refresher programs on geriatric dentistry need to be organized to improve the knowledge, attitudes, and performance of dentists.

## Data Availability

The data that support the findings of this study are available from the corresponding author upon reasonable request.
